# The Vaccination Papers Needed

**Published:** 1898-06

**Authors:** E. W. Sawyer

**Affiliations:** Chicago


### THE VACCINATION PAPERS NEEDED.

Chicago, May 13th, 1898.

Dear Dr. James:—In the March number of The Homœopathic Physician, page 95, is something surprising. That old-school physicians should object to this literature we naturally expect, but the homœopathic physicians who object surely need more light. From my standpoint vaccination is the crowning curse of this century; that it lays the foundation for innumerable diseases, and is one of the elements that enter into cancer, and it explains to a great extent the rapid spread of that hideous disease, I know if I know anything. I do not know whether our homœopathic brethren our aware of the fact that some forms of leprosy are also rapidly spreading in this country. To me it is an alarming fact, and I attribute its spread almost wholly to vaccination. These may be small matters and unworthy the attention of the profession, but I do not so see it.

Very respectfully yours,

E. W. Sawyer.

[Dr. Sawyer is a Professor in Dunham Medical College.—Ed.]

